# Caffeine-folic acid-loaded-chitosan nanoparticles combined with methotrexate as a novel HepG2 immunotherapy targeting adenosine A2A receptor downstream cascade

**DOI:** 10.1186/s12906-023-04212-4

**Published:** 2023-10-27

**Authors:** Alaa Hamed, Doaa Ghareeb, Tarek M. Mohamed, Mahmoud Hamed, Mohammed S. Nofal, M. Gaber

**Affiliations:** 1 Biochemistry Division, Chemistry Department, Faculty of Science, Tanta University, Tanta, 31527 Egypt; 2 Bio-Screening and Preclinical Trial Lab, Biochemistry Department, Faculty of Science, Alexandria University, Alexandria, Egypt; 3 Pharmaceutical Services Center, Faculty of Pharmacy, Tanta University, Tanta, 31111 Egypt; 4 Center of Excellency for Drug Preclinical Studies (CE-DPS), Pharmaceutical and Fermentation Industries Development Centre, City of Scientific Research and Technological Applications (SRTA-City), New Borg El-Arab, Alexandria, Egypt; 5 Chemistry Department, Faculty of Science, Tanta University, Tanta, 31527 Egypt

**Keywords:** Target delivery, Immune suppression, CD39, CD 73, Combination, Apoptosis

## Abstract

**Background:**

Methotrexate (MTX) is a common chemotherapeutic drug that inhibits DNA synthesis and induces apoptosis. Treatment with MTX increased CD73 expression, which leads to higher levels of extracellular adenosine. Adenosine levels are also high in the tumor microenvironment through Cancer cells metabolism. That promotes the survival of cancer cells and contributes to tumor immune evasion through the Adenosine 2a Receptor. A2A receptor antagonists are an emerging class of agents that treat cancers by enhancing immunotherapy, both as monotherapy and in combination with other therapeutic agents. Caffeine is an adenosine receptor antagonist. Herein, we demonstrate the ability of a novel well prepared and characterized nano formula CAF-FA-CS-NPs (D4) for A2aR blockade when combination with MTX to improve its antitumor efficacy by enhancing the immune system and eliminating immune suppression.

**Methods:**

CAF-FA-CS-NPs (D4) were prepared and characterized for particle size, loading efficiency, and release profile. Molecular docking was used to validate the binding affinity of caffeine and folic acid to A2A receptor. The effects of the nano formula were evaluated on human liver cancer cells (HepG2), breast cancer cells (MCF-7), and MDA-MB-231, as well as normal human cells (WI-38). Different combination ratios of MTX and D4 were studied to identify the optimal combination for further genetic studies.

**Results:**

Molecular docking results validated that caffeine and folic acid have binding affinity to A2A receptor. The CS-NPs were successfully prepared using ionic gelation method, with caffeine and folic acid being loaded and conjugated to the nanoparticles through electrostatic interactions. The CAF loading capacity in D4 was 77.9 ± 4.37% with an encapsulation efficiency of 98.5 ± 0.37. The particle size was optimized through ratio variations. The resulting nanoparticles were fully characterized. The results showed that (D4) had antioxidant activity and cytotoxicity against different cancer cells. The combination of D4 with MTX (IC50 D4 + 0.5 IC50 MTX) resulted in the downregulation of *Bcl-2*, *FOXP3*, *CD39*, and *CD73* gene expression levels and upregulation of *Bax* and *A2AR* gene expression levels in HepG2 cells.

**Conclusions:**

This study suggests that CAF-FA-CS-NPs (D4) in combination with MTX may be a promising candidate for cancer immunotherapy, by inhibiting A2aR signaling and leading to improved immune activation and anti-tumor activity of MTX.

## Background

Cancer is a complex disease that is difficult to be diagnosed or treated. Cancer treatment involves a combination of therapies related to the patient's specific cancer type and stage, including surgery, radiation therapy, chemotherapy, immunotherapy, targeted therapy, and hormonal therapy [1]. Chemotherapy is a common cancer treatment but its effectiveness is limited by its toxicity to normal cells [2]. So, regular monitoring for this type of treatment is important to assess its effectiveness and manage possible side effects [3].

Methotrexate (MTX) is known as a traditional powerful chemotherapy which is the most commonly used first-line drug among clinical chemotherapeutic strategies [4]. MTX is frequently used to treat various cancers, such as acute lymphoblastic leukemia, head and neck cancer, lung cancer, and breast cancer. It can be administered alone or in combinations [5]. Its mechanism of action involves inhibiting the production of tetrahydrofolate (THF), an essential coenzyme responsible for DNA synthesis, repair, and replication. MTX targets dihydrofolate reductase (DHFR), blocking the synthesis of THF and disrupting cell proliferation [6]. This metabolic imbalance leads to a reduction in the growth of cancerous cells [7–9]. Many studies highlighted the importance of MTX encapsulation strategies for improving its efficacy in cancer treatment [10]. Furthermore, MTX exhibits immunosuppressive properties that prove beneficial in the treatment of severe inflammation and autoimmune diseases such as arthritis, psoriasis, and myasthenia gravis.

The use of MTX results in an elevation of adenosine levels outside of cells. This increase in adenosine has been observed to have an anti-inflammatory effect through the A2A and A3 adenosine receptors [11, 12].

Methotrexate's anti-inflammatory properties are mediated by the enzyme ectonucleoside triphosphate diphosphohydrolase-1 (CD39/ENTDP1), which produces extracellular ADO through ATP metabolism [13]. Cancerous cells also activate CD39 and CD73 triggering vascular angiogenic responses that aid in the growth of tumors [14, 15]. CD39 and CD73 are highly expressed and active of in several blood or solid tumors [16]. The CD39/CD73 complex participates in the process of tumor immune escape, via pericellular generation of adenosine that has role in suppressing T cell activation [17].

Accumulation of extracellular adenosine stimulate A2A receptor cascade leads to cancer progression via supporting of angiogenesis [18], promotion of tumor cell migration [19] and immunosuppressive cellular responses [20–22]. Extracellular adenosine binds to A2A receptor, causing Gs-protein coupled response, accumulating intracellular cAMP, upregulating inhibitory cytokines such as TGF-β and PD-1 [23]. These molecular targets is therapeutically exploited for cancer treatment [17]. So, research has focused on targeting tumor-associated adenosine signaling to enhance the immune response to malignancy [24]. A2AR antagonists block the adenosine A2A receptor and show promise in cancer immunotherapy [25].

A number of clinical trials are investigating adenosine pathway inhibitors [26]. Interestingly, A2AR antagonists like ZM241385 [27]and SCH58261 [28]can effectively reduce primary tumor growth, even in a T cell-independent manner [29]. Other A2AR antagonists CPI-444 [30],T NIR178 [31], AZD4635 [32] and PFB509 [33] Were administered as both monotherapy and in combination with other anticancer factors demonstrated improved antitumor efficacy. Numerous cancer immunotherapeutic approaches, including monoclonal antibodies, immune checkpoint inhibitors, cancer vaccines, and cell-based therapies, have shown effectiveness in a wide range of patients [34–36]. 

Caffeine (CAF) is a well-known antagonist of the adenosine A2A receptor [37, 38]. CAF is a naturally occurring alkaloid, included in food and beverage, pharmaceuticals, cosmetics, and dietary supplements [39]. Combining caffeine with various anticancer medications has improved their cytotoxic effects [40]. Apoptosis and DNA damage repair pathways are two molecular pathways linked to CAF's anticancer activity [41]. Different nano systems have been used to formulate CAF, including liposomes [42], ethosomes [42] solid lipid nanoparticles [43], and polymeric nanoparticles made from synthetic polymers such polycaprolactone [44]. Chitosan (CS) has been suggested as one of the materials utilized to make caffeine nanocarriers [45, 46].

A nanocarrier (Nc) is used to locally deliver cytotoxic compounds to reduce side effects and advance medical results [47]. Materials with nanoscale properties can be used to deliver medicinal drugs to specific targeted locations in a controlled manner or as diagnostic tools [48]. Since chitosan (CS) and its derivatives have strong biocompatibility, biodegradability, and nontoxic qualities, they are employed in the domains of medicine, food, feed, chemical, agriculture, environmental protection, and biotechnology [39, 49]. CS-NPs that are crosslinked by sodium tripolyphosphate (TPP) are controlled release carriers that increase the stability of the medication, deliver it to specific areas, and increase its bioavailability [50–52]. It is utilized as a carrier for a variety of medications [53, 54], including herbal extracts [55], antibacterial medicines [56], antiviral drugs [57], and anticancer agents [58].

Nanoparticles can target cancer cells in two ways: passively and actively [59]. Both methods enhance the precision and effectiveness [59, 60]. Passive targeting occurs when the size of the particles and EPR effect allow them to passively diffuse through the cell membrane and target cancer cells [61]. On the other hand, active targeting involves attaching ligands to the surface of nanoparticles to actively seek out their target and accumulate in cancer cells, leading to more precise and effective drug delivery [62].

Folic acid (FA), one of the most widely used ligands, has a high affinity for its folic acid receptor (FR) that are overexpressed on the membrane of most of cancer cells [63]. Because of this, delivery of folic acid and folate conjugates to FR-positive tumor cells was significantly improved. In the treatment of carcinomas such as lung, breast, ovarian, colon, renal, mesothelioma, etc., this nano system functions as a perfect carrier when FA is coupled with an anticancer agent such as (5-fluorouracil, doxorubicin, cisplatin, GEM, vincristine, PTX, methotrexate, etc.) [64–66]. 2FA and chitosan can be combined through electrostatic interaction between the cationic amino group in chitosan and the anionic carboxylic group in FA to generate safe and efficient FA-CS-NPs [67].

Methotrexate (MTX) is FDA-approved for treating a range of cancers, including ALL, brain tumors, breast cancer, hepatoma, lung cancer, lymphomas, and more. It can be used alone or in combination with other drugs for effective treatment [68–70]. High-dose or prolonged use of MTX in cancer treatment often lead to significant side effects [70].

**Fig. 1:**
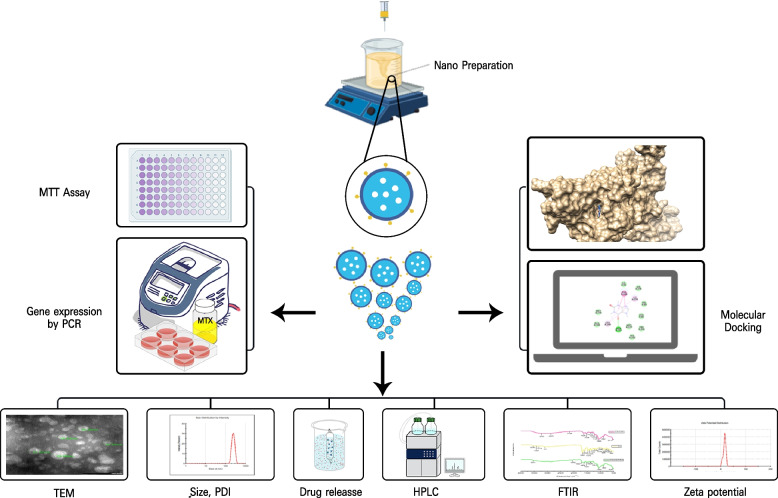
A graphical abstract summarizing the study

Considering our previous observations, this study aimed to develop and characterize a novel formula CAF-FA-CS-NPs(D4), CS acting as a nanocarrier for caffeine and folic acid facilitating active targeting. The primary goal was to utilize these nanoparticles to deliver caffeine to block adenosine receptors that are overexpressed in cancer cells and because of MTX intake. By combining caffeine with methotrexate (MTX), the study aimed to enhance the immune system, eliminating immune suppression, and promoting improved recognition of cancer cells. Additionally, each ingredient in the formula has its own anticancer properties. The cytotoxicity effect has been evaluated on normal and cancer cells. The best combination ratio between D4 and MTX has been estimated to target A2A receptor and enhance the effect of MTX by reducing its immune suppression effect. Therefore, this effect was tracked by measuring on the apoptotic markers, Foxp3, CD73, CD39 and A2A receptor gene expression. Figure 1 summarizes our work.

## Methods

### Materials

Sodium tripolyphosphate (TPP), sodium hydroxide and by 2,2-Diphenyl-1-picrylhydrazyl (DPPH) were bought from Sigma-Aldrich, while caffeine, M. wt. 194.19 g/mol, was purchased from Loba chemie, India. Chitosan with > 90% purity and degree of deacetylation of 90% was purchased from Bio Basic Inc., Canada. Research-Lab Fine Chem Industries supplied the folic acid (Molecular Weight: 441.40). From Friendemann Schmidt, acetic acid glacial (Grade AR, CH3COOH, M. Wt. 60, 05 g/mol) was obtained. Fischer Scientific Korea Ltd. provided high-performance liquid chromatography (HPLC)-grade acetonitrile (Seoul, Korea). The cell lines utilized in the study were provided by the Pharmaceutical & Fermentation Industries Development Center in SRTA-City and obtained from the American Type Culture Collection (ATCC). The specific cell lines used were MCF7 (ATCC HTB-22), MDA-MB-231 (ATCC CRM-HTB-26), Hep G2 [HEPG2] (ATCC HB-8065), and WI-38 (ATCC CCL-75). The other chemicals were of an analytical grade.

### Preparation of CS-NPs, CAF-CS-NPs, FA-CS-NPs, and CAF-FA-CS-NPs

#### Preparation of (CS-NPs)

Chitosan nanoparticles were synthesized by ionic gelation which involves the electrostatic interaction between chitosan's amine groups (NH3 +) and sodium tripolyphosphate's phosphate groups (PO4-) [71]. Chitosan (1 mg/ml) was dissolved in 1% (v/v) acetic acid (pH 3.3) and left under high stirring (1000 rpm/min) for 1 h and filtrated through 0.45 μm filter. TPP (0.625, 1.25 and 1.87 ml) as a physical cross linker was dissolved separately in deionized water and added dropwise to 5 ml of 0.25, 0.5 and 0.75 mg/ml chitosan solution, respectively, under magnetic stirring (1000 rpm) at room temperature. The colloidal suspension turned opalescent and was stirred for 1 h followed by centrifugation at 10,000 rpm for 45 min. The pellet was washed the times 10 mint for each and freeze dried to obtain powders.

#### Preparation of (CAF-CS-NPs)

The ratios of (CAF/CS; 0.25%, 0.25%, and 0.75% w/w) were used to load various caffeine concentrations into chitosan nanoparticles. Depending on the ideal nano-size and polydispersity (PDI), an optimum formulation was selected [72].

#### Preparation of (FA-CS-NPs)

Two strategies for conjugating folic acid with chitosan nanoparticles were applied. The first one, folic acid was dissolved in TPP solution (1.5 mg/mL) before dropping into chitosan solution (0.75 mg/mL). In the second method 2 mg folic acid was dissolved in 10 mL milli Q water, then 100 µL of 1 M NaOH was added for complete dissolution. 0.375, 0.75 and 1.25 mg/mL of folic acid was mixed with TPP and dropped into of CS solution (0.75 mg/mL). The second method gave better results of nano size and PDI [67, 73].

#### Preparation of (CAF-FA-CS-NPs)

Based on optimized ratios and concentrations from previous experiments, CAF-FA-CS NPs were formed spontaneously by adding an aqueous solution of TPP and folic acid drop by drop into a solution of chitosan and caffeine under magnetic stirring at 0 °C for 1 h [74].

### Characterization of different formula

#### Size analysis and zeta potential

CS-NPs were synthesized, and dynamic light scattering was used to evaluate the particle size and PDI of the unloaded and drug-loaded samples (Nano ZS, Malvern, and Worcester-shire, UK) [75].

#### Transmission electron microscopy analysis

Transmission electron microscopy (TEM) (JEOL, JEM 1400, Tokyo, Japan) operating at an acceleration voltage of 80 kV was used to determine the morphology (shape and size) of the prepared CS-NPs formulations [76, 77].

#### Fourier Transform Infrared spectroscopy (FT-IR) analysis

Samples that had previously been lyophilized were investigated for their infrared spectrum. With the help of FTIR, the chemical structures of CS, FA, CAF, CS-NPs, CAF-CS-NPs, FA-CS-NPs, and CAF-FA-CS-NPs were studied. All spectra were obtained from 4000 to 400 cm^−1^ at room temperature and subtracted from the baseline of black background [78].

#### Encapsulation efficiency and loading capacity

The encapsulation and loading efficiencies of the nanoparticles were determined by first separating them from the aqueous medium by ultracentrifugation at 10,000 r/min for 45 min at 4 C. Various standard solutions of caffeine and Folic acid were prepared and subjecting to reversed phase HPLC [72].

#### Assessment of Drug Release

Drug release was carried out using the dialysis bag method (visking® 28 mm, MWCO 12,000–14,000; Serva, Heidelberg Germany).Samples were filtered through 0.45 μm filter and analyzed HPLC [79].

#### HPLC analysis

**Fig. 2:**
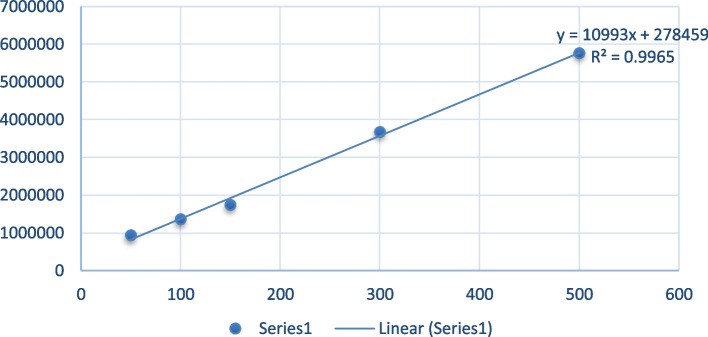
Standard curve of Caffeine

**Fig. 3:**
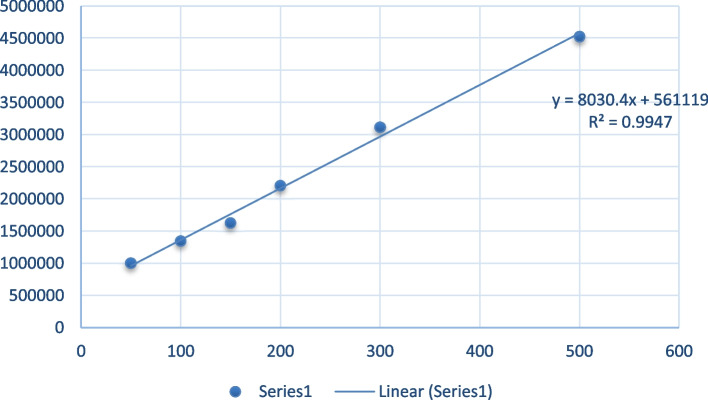
Standard curve of Folic acid

Reversed phase HPLC method(HPLC; Shimadzu HPLC LC-2010, Japan) was used to measure the concentration of caffeine and folic acid [80]. The column used was C18 column (Shimadzu, 4.6 mm × 250 mm, 5µ. M). The mobile phase consists of 0.1% trifluoro acetic acid (A), Acetonitrile (B). 20 µL of sample was injected into the liquid chromatography with a flow rate of 1.0 mL/min. UV detection is performed at 280 nm. The linearity is measured from the calibration curve of standard solutions containing 0.1–1.0 mg/mL (*n* = 10) of caffeine. The precision and accuracy of this method are expressed as coefficient of variation (%CV) and relative standard error (% E) in accordance with FDA guidelines. Figures 2 and 3 shows the standard curve of caffeine Folic acid respectively.

#### Molecular docking

Auto dock Vina through the LINUX command line to run all the docking. The X-ray crystal structure of A2A in complex with NGI was downloaded from the protein database PDB ID 4UHR. [81]. First, we used Auto Dock tools to optimize hydrogen and charges of the receptor. The coordinates of the binding site of the A2A enzyme were constructed using the grid box function Table 1 where the co-crystallized NGI is bound. As part of the docking validation, the X-ray coordinates of the co-crystallized ligands were obtained by redocking to the given active site. The previous step yielded an RMSD value of 1.215 Å between the co-crystal and docking poses, indicating a valid docking protocol. The compounds caffeine and folate were then docked into the A2A binding site. Finally, to analyze the docking results, 2D and 3D interaction diagrams were generated by BIOVIA Discovery Studio Visualizer and UCSF Chimera, respectively.

**Table 1:** Co-ordinates of the binding pocket for the target protein

	**Size**	**Centre**
**X dimensions**	20	-18.638
**Y dimension**	26	7.011
**Z dimensions**	54	-43.468

#### DPPH radical scavenging activity

The DPPH radical scavenging activity of compounds was measured using a modified version of the assay proposed by [82].

#### Cell Viability assays

To examine cell viability, human liver cancer cells (HepG2), breast cancer cells (MCF-7 and MDA), and normal human cells (WI-38) were used [1]. At a density of 5 × 10^4^ cells per mL, 150 μL of media containing cells was added to the 96-well plate, which was then incubated for 24 h at 37 °C with 5% CO2. Cells were exposed to suspension of CS-NPs, CAF-CS-NPs, FA-CS-NPs, CAF-FA-CS-NPs(D4), caffeine, folic acid, and caffeine/ folic acid mixture(D7) at different amounts of 31.25, 62.5, 125, 250, 500 and 1000 μg/mL. 150 *μ*L of medium containing cells were added to the 96-well plate at a density of 5 × 10^4^/ mL and incubated for 24 h at 37 °C under 5% CO_2_. Cells were subjected to suspension of CS-NPs, CAF-CS-NPs, FA-CS-NPs, CAF-FA-CS-NPs, caffeine, folic acid, and caffeine/ folic acid combination at concentrations of 31.25, 62.5, 125, 250, 500 and 1000 μg/mL. After 72 h, 20 μL MTT with concentration of 5 μg/mL was added into 96-well plate and incubated for 4 h at 37 °C. After removing the cultural supernatant, 150 μL DMSO was added to each well and stirred for 30 min. Using a microplate reader (Metertech AcuuReader M965), the solution's absorbance was determined at a wavelength of 570 nm. The cell viability was calculated by taking the ratio of the treated cell culture's absorbance to that of the untreated control and multiplying it by 100 to get a percentage. This percentage represents the cell viability, or the percentage of control (percentage of control, %).

#### Reverse transcription-quantitative polymerase chain reaction (RT-qPCR)

Real-time PCR with SYBR Green was used to measure the expression of target genes in HepG2 cells treated with different formulations and ratios Table 2. The expression was measured by amplifying the isolated cDNA using gene-specific primers Table 3 and a 2X Maxima SYBR Green/ROX qPCR Master Mix, following the manufacturer's protocol (Thermo scientific, USA, # K0221) and gene specific primers. The primers were designed using the web-based tool Primer 3 and were checked for uniqueness in the template sequence using BLAST. B-actin was used as the internal reference [83].

**Table 2:** Different combination ratios between MTX IC50 and (D4& D7) formulas each alone

IC50	0.5 MTX	1 MTX	2 MTX
**0.5 D4**	0.5 + 0.5	0.5 + 1	0.5 + 2
**1 D4**	1 + 0.5	1 + 1	1 + 2
**2 D4**	2 + 0.5	2 + 1	2 + 2
**0.5 D7**	0.5 + 0.5	0.5 + 1	0.5 + 2
**1 D7**	1 + 0.5	1 + 1	1 + 2
**2 D7**	2 + 0.5	2 + 1	2 + 2

**Table 3:** Forward and reverse primer sequence for candidate genes

Gene	Forward primer (^/^5 ––– ^/^3)	Reverse primer (^/^5 ––– ^/^3)
***Bax***	TGCTTCAGGGTTTCATCCAG	GGCGGCAATCATCCTCTG
***Bcl-2***	AGGAAGTGAACATTTCGGTGAC	GCTCAGTTCCAGGACCAGGC
***FoxP3***	TCATCCGCTGGGCCATCCTG	GTGGAAACCTCACTTCTTGGTC
***CD73 (NT5E)***	ATTGCAAAGTGGTTCAAAGTCA	ACACTTGGCCAGTAAAATAGGG
***CD39 (ENTPD1)***	GAAGGTGCCTATGGCTGGATTAC	TGTTGGTCAGGTTCAGCATGTAG
***A2AR (ADO)***	AGCTGAAGCAGATGGAGAGC	AGGGATTCACAACCGAATTG
***B-actin***	GGATGCAGAAGGAGATCACTG	CGATCCACACGGAGTACTTG

### Statistical analysis

The one-way ANOVA analysis of variance was used by GraphPad Prism 8. Data was presented as mean ± standard deviation of different independent experiments. Statistical differences were considered at *p* < 0.05.

## Results and discussion

### Size analysis and zeta potential

**Fig. 4:**
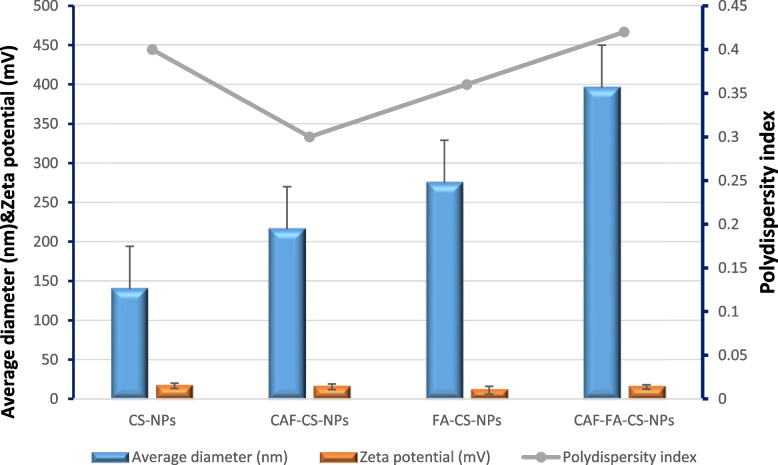
Mean diameter (nm), Zeta potential (mV) and Polydispersity index of nanoparticles prepared

**Fig. 5:**
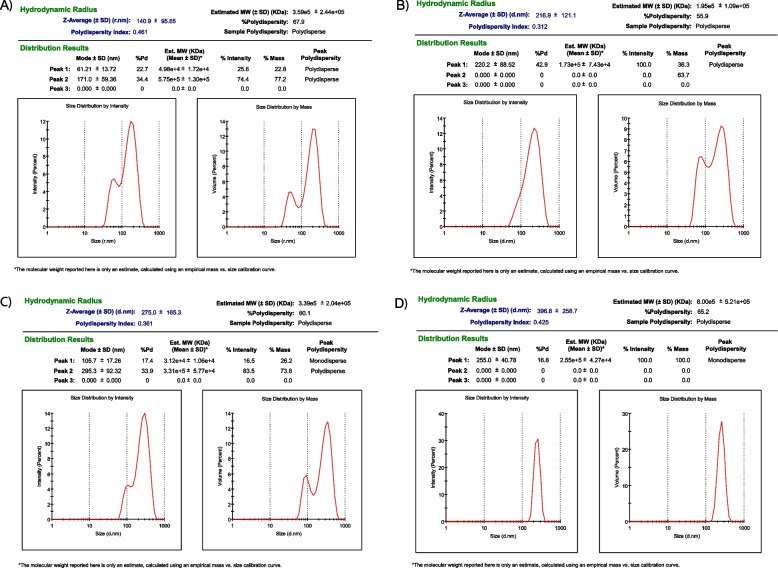
The hydrodynamic particle size diameter (nm), PDI of the optimal formulations of CS-NPs (**A**), CAF-CS-NPs (**B**), FA-CS-NPs (**C**), and CAF-FA-CS-NPs (**D**)

The particle size of chitosan nanoparticles was altered by changing the concentrations of TPP (1.5, 1.6, 2 mg/mL) and chitosan (0.25, 0.5, 0.75 mg/mL). The optimal condition was found to be 0.75 mg/mL chitosan and 1.5 mg/mL TPP, resulting in a particle size of 140 ± 95.65 nm Table 4 and Figs. 4 and 5. An excess of TPP caused more intramolecular crosslinking and larger particle size with unequal distribution [84], while insufficient TPP resulted in less chitosan formation and difficulty in polymerizing and loading drugs [85].

**Table 4:** Summarized data for Mean diameter (nm), Zeta potential (mV) and Polydispersity index of nanoparticles prepared

Parameters	CS-NPs	CAF-CS-NPs	FA-CS-NPs	CAF-FA-CS-NPs
**Mean diameter (nm)**	140 ± 95.65	216 ± 121.1	275 ± 165.3	396 ± 258.7
**Zeta potential (mV)**	16.6 ± 3.46	15.4 ± 3.58	11 ± 4.99	15.2 ± 2.78
**Polydispersity index**	0.4	0.3	0.36	0.42

The size of CAF-CS-NPs increased from 312 to 341 nm (Table 4 and Figs. 4 and 5) when the CAF concentration was increased from 0.25 to 0.75 mg/mL, due to the formation of denser complexes and larger self-assembled nanoparticles. However, the addition of folic acid (FA) resulted in a size of 396 ± 258.7 nm. This small size allows for improved targeted delivery to cancer cells through binding to overexpressed folate receptors, leading to increased accumulation within the tumor region. The CAF-FA-CS-NPs demonstrated both active and passive targeting, folic acid facilitate active targeting and the small size helps for passive targeting through the enhanced permeability and retention (EPR) effect [86, 87]. The low polydispersity index (PDI) value of 0.42 of (D4) Table 4 indicates that the particles are uniform in size and are unlikely to aggregate, resulting in more consistent and predictable properties as favorable particle size distribution (PDI < 0.5) [88, 89].

**Fig. 6:**
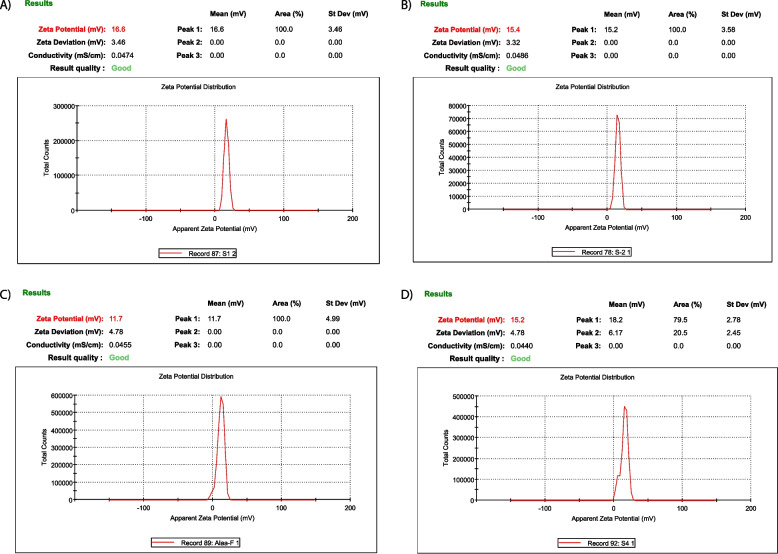
The hydrodynamic zeta potential (mV) of the optimal formulations of CS-NPs (**A**), CAF-CS-NPs (**B**), FA-CS-NPs (**C**), and CAF-FA-CS-NPs (**D**)

The zeta potential of the prepared CS-NPs was 16.6 ± 3.46mV. The zeta-potential is used to determine the stability of colloidal systems. A high positive zeta potential corresponds to the repulsive interaction between nanoparticles aimed at preventing the agglomeration of NPs [90]. After loading of caffeine (CAF-CS-NPs), zeta potential was 15.4 ± 3.58 mV. For FA-CS-NPs, zeta potential decreased to 11 ± 4.99 mV. The addition of folic acid and/or caffeine led to a decrease in electrical stability (lower zeta potential) and an increase in particle size, as demonstrated in Table 4 and Figs. 4, 5 and 6. The decrease in zeta potential is believed to be caused by the neutralization of positive charges from the chitosan molecules' amino groups when they come into contact with negatively charged folic acid. CAF-FA-CS-NPs had zeta potential of 15.2 ± 2.78 mV (Table 4 and Figs. 4 and 6) which ensure the optimum stability of all the suspensions [91]. Modified nanoparticles with folate demonstrated an excellent targeted delivery capability [92].

Increasing the mass ratio of chitosan (CS) caused the nanoparticles to have a higher positive charge, likely due to an increase in the number of free amino groups on their surface. A positive zeta potential makes it easier for the particles to pass through the negatively charged cell membrane of cancer cells and also shows the stability of the particles in aqueous solutions [93, 94].

### Fourier Transform Infrared Spectroscopy (FT-IR) analysis

**Fig. 7:**
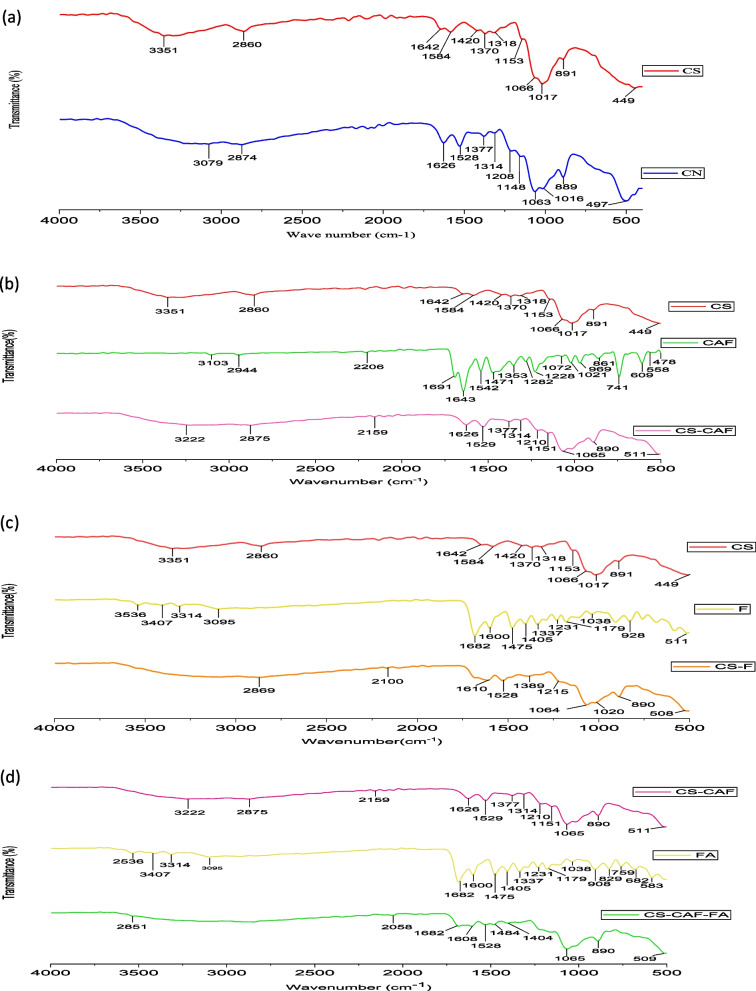
The FTIR spectra of (CS&CS-NPs (**A**)), (CS&CAF&CAF-CS-NPs (**B**)), (CS&FA&FA-CS-NPs (**C**)), (CAF-CS-NPs &FA&CAF-FA-CS-NPs (**D**))

The CONH2 and NH2 groups, respectively, are responsible for the characteristic CS peaks at 1642 cm^−1^ and 1584 cm^−1^ [95] Fig. 7a. In the FTIR analysis of (CS-NPs) Fig. 7a, a shift was observed in the peaks at 1642 cm^−1^ and 1584 cm^−1^, attributed to the CONH2 and NH2 groups, respectively. This shift was found to be caused by the interaction between the NH3 + groups of chitosan and the phosphate groups of TPP. The FTIR spectra also revealed the presence of -CH2 wagging at 1318 cm^−1^, as well as a peak at 1208 cm^−1^ typical of P = O stretching vibrations from the phosphate groups. These findings are similar to those reported in previous studies of TPP treated with chitosan nanoparticles [96–98].

Based on Fig. 7b, The FTIR spectrum of caffeine displayed key peaks associated with the alkyl group (-CH2) at 741 cm^−1^, the amide group III (-CN) at 1471 cm^−1^, and the amine group I (-NH) at 1691 cm^−1^. Upon loading caffeine into CS-NPs, the –OH peak (3222 cm^−1^) broadened, suggesting an increased bonding interaction between CS and caffeine. Additionally, a new peak appeared at 1210 cm^−1^, indicating the presence of a carbonyl group (C = O) in caffeine. The peak at 1318 cm-1 in the CS spectra shifted to 1377 cm^−1^ in the CAF-CS-NPs spectra, indicating an interaction between the C = O group of caffeine and the primary amide group of CS [45, 46]. Hence, the results suggest that caffeine was successfully loaded into the CS-NPs.

According to Fig. 7c, the FTIR spectrum of folic acid (FA) displays an OH peak at 3536 cm^−1^. Other notable absorption peaks are found at 3407, 1682, 1600, and 1475 cm-1, representing vibrations of N–H, C = O, an amino group in the pteridine ring, and C = C or C = N of FA [99]. The results of the FTIR analysis of the FA-CS-NPs spectrum suggest that folate was successfully conjugated to chitosan. The spectrum showed not only the characteristic bands of chitosan, but also the presence of new bands, such as the amide band at 1610 cm-1 and the N–H bending in the second amine at 1528 cm^−1^, which indicate the conjugation of the -COOH of folate to the amino group of chitosan [66, 100]. It can be seen that the absorption peak at 2869 cm^−1^ became stronger due to the overlapping of the vibration of OH and N–H functional group [101, 102]. In the FTIR spectrum of the FA-CS-NPs, the shift of the -NH2 bending vibration peak from 1642 cm-1 to 1610 cm^−1^ indicates the connection between TPP and the ammonium ion of the FA-CS-NPs. Additionally, the broadening and strengthening of the 2869 cm^−1^ peak, due to the overlapping of the vibration of the OH and N–H functional groups, suggests that the inter- and intra-molecular interactions are enhanced in the FA-CS-NPs because of the TPP groups linking with the ammonium group of the FA-CS-NPs [101–103].

The FTIR spectrum Fig. 7d showed that CAF-CS-NPs displayed the N–H stretching vibration peak at 1626 cm^−1^, which had shifted to 1608 cm^−1^, indicating the interaction between the two components. Furthermore, the wider peak at 2851 cm^−1^ indicates that the linking of the sodium tripolyphosphate groups with the ammonium group of the folic acid-chitosan conjugate has strengthened the inter- and intra-molecular actions within the nanoparticles [104]. The FTIR spectra of CAF-CS-NPs showed a peak at 1529 cm^−1^, which was attributed to the N–H deformation vibration, suggesting a connection between the sodium tripolyphosphate and the ammonium ion of the folic acid-chitosan conjugate. However, this peak shifted to 1528 cm^−1^ in CAF-FA-CS-NPs, indicating an interaction between FA and CAF-CS-NPS forming (D4) [64].

### Transmission electron microscopy

**Fig. 8:**
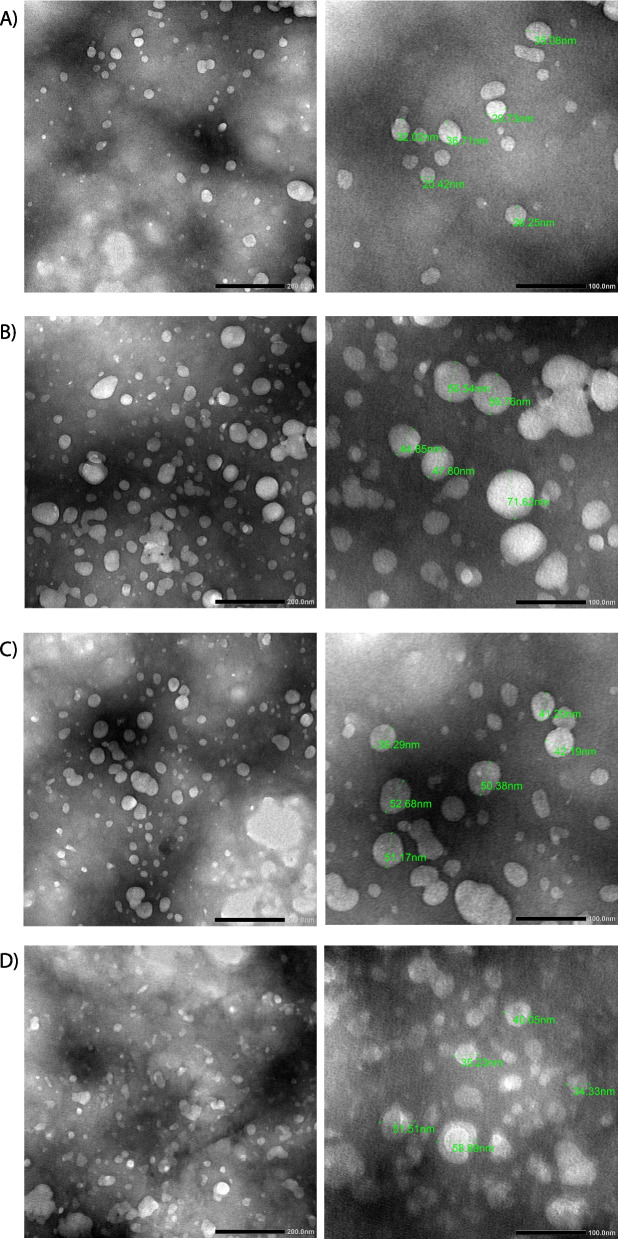
Transmission electron microscopy of the optimal formulations of CS-NPs (**a**), CAF-CS-NPs (**b**), FA-CS-NPS (**c**), and CAF-FA-CS-NPs (**d**). (Scale bar is 200 nm and 100 nm)

The transmission electron microscopy results indicate that the nanoparticles have a spherical shape and uniform size distribution, ranging from 25 to 90 nm, as demonstrated in Fig. 8. The optimized formulation results in a uniform particle size distribution. The size is smaller than the one obtained from the dynamic light scattering method, which is caused by the lack of the hydration shell during the TEM particle size determination as The DLS technique measures the hydrodynamic diameter of nanoparticles (indicative of the apparent size of the dynamic hydrated/solvated particle) in their dispersed state in water [105].

### Encapsulation efficiency, loading capacity and In Vitro Drug Release

Desirable administration systems aim to prolong the therapeutic effect of drugs after administration. Sustained release of caffeine can lengthen its duration of action, keep drug levels consistent, reduce systemic side effects, lower dosing frequency, and increase efficacy [106]. To determine the release of Caffeine from D4, in vitro release was simulated in phosphate-buffered saline (pH 7.4) which used to mimic the physiological cell conditions [107]. The loading capacity of nanocarrier was around 40% with 97- 99% loading efficacy for caffeine and around 33% with loading efficacy 78–80% for Folic acid Table 5. Moreover, the drug release study showed that 69 to 77.9% of the caffeine were released after 3.5 h and 53% for folic acid at the same time, Table 6. The loading capacity of blank nano-carrier CAF-CS-NPs was around 37% with 95—97% loading efficacy for caffeine. The loading capacity of blank nanocarrier FA-CS-NPs was around 35% with 96—99% loading efficacy for Folic acid Table 5. These blank nanoparticles also could be used as an antidote [108]. Folic acid, which is commonly used as a ligand to target cancer cells, can be integrated into chitosan-based drug delivery systems to improve their targeting ability [109].

**Table 5:** Encapsulation efficiency, loading capacity and Wight Yield % of CAF and FA

CAF-CS-NPs			FA-CS-NPs				CAF-FA-CS-NPs			
**CAF EE%**	**CAF LC%**	**CAF Wight Yield %**	**FA EE%**	**FA LC%**	**FA Wight Yield %**	**CAF EE%**	**CAF LC%**	**FA EE%**	**FA LC%**	**Mean of CAF, FA LC%**
96.02 ± 0.4	45.8 ± 1.04	48%	98.6 ± 0.32	35.1 ± 0.76	50 ± 0.82%	98.5 ± 0.37	39.5 ± 0.51	77.8 ± 0.76	32.7 ± 0.25	36.5 ± 0.52

**Table 6:** In Vitro Drug Release of Caffeine and Folic acid

	**Release**	
Time	**Caffeine**	**Folic acid**
**1 h**	36.8 ± 3.6%	2.15 ± 0.37%
**2 h**	52.1 ± 3.04%	3.3 ± 0.3%
**3 h**	77.9 ± 4.37%	53 ± 3.9%

### Molecular modeling studies

**Fig. 9:**
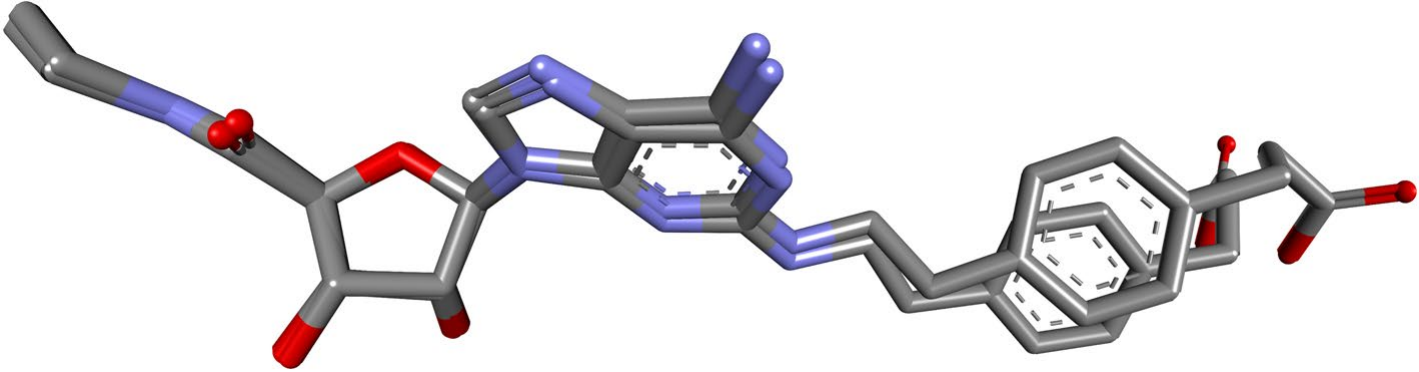
Validation of docking method by superimposing NGI present in the crystal structure of 4UHR and that after redocking the same

**Fig. 10:**
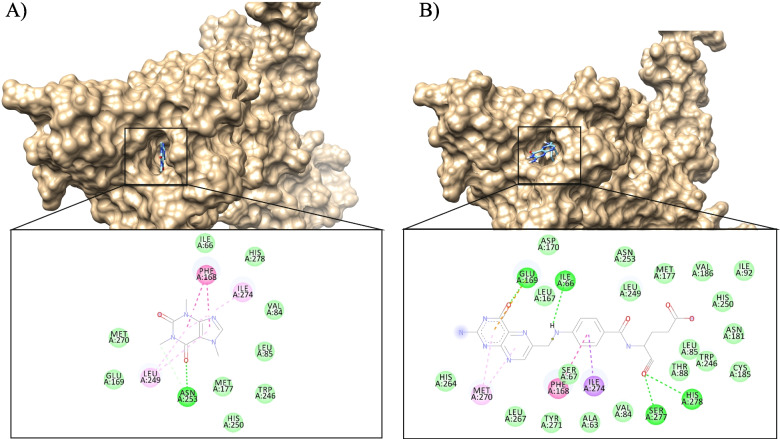
2D and 3D structure for Protein ligand interaction between A2A and Caffeine (**A**) and Folic acid (**B**)

Molecular docking is a computer-aided drug design tool that plays a significant role in drug discovery and reduces the cost and time required for this process. The redocking of the co-crystallized ligand NGI was performed to validate the docking protocol, and the RMSD value was less than 2 Å compared to the co-crystallized ligand, indicating a valid docking protocol Fig. 9. The results of docking Caffeine and folic acid into the active site of A2A showed high affinity for the receptor and both ligands fit perfectly within the active site Table 7. The 2D and 3D structure of selected interactions between A2A with Caffeine and Folic Acid are supplied in Fig. 10.

**Table 7:** Binding affinity score of the ligands and their interaction with the adenosine A2A receptor

Ligand	Vina score (Kcal/Mol)	Residues involved in hydrogen bonds interactions	Residues involved in hydrophobic Alky- Alkyl interactions	Residues involved in pi-interactions
**CGS-21680**	-10.9	ASN 253(2), GLU 169, HIS 278(2), HIS 250, LYS 153	PHE 168	ALA 89, LEU 85, CYS,185
**Caffeine**	-6.5	ASN 253	LEU 249 (2)	PHE 168 (2)
**Folic Acid**	-9.8	GLU 169, ILE 66, HIS 278, SER 277	PHE 168	MET 270(2)

Caffeine is a known antagonist of the adenosine receptor, blocking this receptor may enhance immunotherapy and improve MTX effect while combination [110].

For the analysis, the tertiary structure of A2A was obtained from the RCSB Protein Databank using the PDBID 4UHR [81]. For comparison CGS-21680 was used as a specific agonist of the adenosine A2A subtype receptor [111].

### Antioxidant activity

**Fig. 11:**
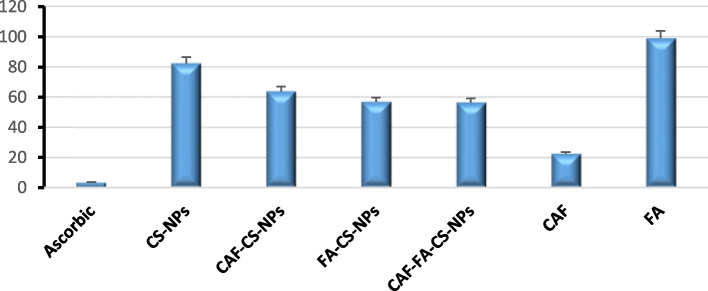
The inhibition concentration values DPPH (IC_50_) value of samples

The antioxidant properties of Chitosan nanoparticles, CAF-CS-NPs, FA–CS-NPs, and CAF-FA-CS-NPs were evaluated and compared the IC50 of the ascorbic acid (3.44 μg/ml). The inhibition concentration values DPPH (IC50) values Fig. 11 showed that caffeine had the strongest DPPH scavenging activity at 22.42 μg/ml while FA had the lowest activity at 99.11 μg/ml Folic acid, a B-group vitamin, can interact with free radicals, contributing to its antioxidant effect [112]. The encapsulation of caffeine in CAF-FA-CS-NPs(D4) significantly enhanced its antioxidant activity compared to caffeine free.

### Cell Viability

The safety assessment of CAF-FA-CS-NPs (D4) and MTX revealed that both compounds were less toxic to normal cells (higher IC50s) when compared to cancer cells (lower IC50s). Particularly, D4 exhibited minimal toxicity towards normal cells (WI-38), as demonstrated in Table 8, indicating its biocompatibility for targeted cancer treatment, aligning with the goal of reducing adverse effects on healthy cells in effective cancer therapies. While *invitro* studies offer initial safety insights, comprehensive in vivo and human clinical trials are essential to thoroughly assess the safety and tolerability of our combination therapy. These trials will identify potential side effects, their severity, and ensure the therapy's safety for human use.

**Table 8:** IC_50_ cytotoxic concentration (μg/ml) on WI, HepG2 cells after 48 h and 72 h incubation and on MDA and MCF7 after 72 h incubation

	**WI** **48 h**	**WI** **72 h**	**HepG2** **48 h**	**HepG2** **72 h**	**MDA** **72 h**	**MCF7** **72 h**
**CS-NPs**	11,114 ± 3.1	5686 ± 2.5	2748 ± 2.5	921.9 ± 2.4	179.7 ± 2.6	144 ± 1.1
**CAF**	7565 ± 4.0	5568 ± 3.8	813.3 ± 2.3	317.7 ± 2.2	389.5 ± 1.5	120.6 ± 0.6
**Folic**	944528 ± 5.2	6070 ± 4.9	743.1 ± 1.3	559.1 ± 5.0	233.5 ± 1.2	261.1 ± 1.0
**D4**	698750 ± 8.5	34,047 ± 4.2	1082 ± 3.0	652 ± 4.0	204.5 ± 0.7	247.2 ± 1.2
**D7**	68962 ± 5.6	3110 ± 5.8	1123 ± 7.8	982 ± 1.1	347.3 ± 1.0	305.1 ± 2.0
**MTX**	725.2 ± 2.9	11.95 ± 0.3	9.764 ± 0.2	8.489 ± 0.2	20.96 ± 0.6	9.22 ± 0.1

The loading capacity of nanocarrier was around 40% for CAF and around 33% for FA Table 5. Cell viability results Table 8 showed a synergistic effect of CAF and FA in D4 formula while administration in HepG2 and MCF-7 cell lines, even for MDA-resistant cell lines [113]. The combination of CAF and FA conjugation with chitosan leads to improved targeting and increased cytotoxicity in cancer cells due to increased expression of folic acid receptors on cancer cells [92]. Theoretically, active targeting based on ligand-receptor recognition may be more effective in human cancer therapy compared to passive targeting alone [114]. The increased cytotoxicity of D4 may be attributed to improved internalization through endocytosis and reduced drug efflux from cells, as well as the high affinity of folate-modified NPs for tumor cells, leading to quick intracellular release of caffeine. Therefore, FA-CS-NPs have great potential as a solution for active targeting drugs to tumors [66].

As reported by Rosendhal et al., MCF-7 cells were more sensitive to the cytotoxic effects of caffeine, with a significant reduction in cell viability at a lower concentration than in MDA-MB-231 cells. This difference was also seen in the evaluation of proliferative capacity, with more evident effects on cell proliferation in MCF-7 cells, indicating their greater susceptibility to the cytotoxic and cytostatic effects of caffeine [115].

The amount of caffeine and folic acid present in a weight of D4 is 3:4 times less than that in D7. This suggests that the nano formulation used in D4 enhances the effect of CAF and FA compared to the mixture in D7. D4 formula has shown a positive effect on HepG2 cells.

Following the determination of IC50 values for both D4 and MTX, we proceeded to design various combination ratios to optimize efficacy in terms of the related genetic expression in HepG2 cells Table 2.

Determining the optimal ratios, correct dosage and administration schedule is essential for achieving therapeutic efficacy to avoid side effects and associated complications.

### Reverse transcription-quantitative polymerase chain reaction (RT-qPCR)

**Fig. 12:**
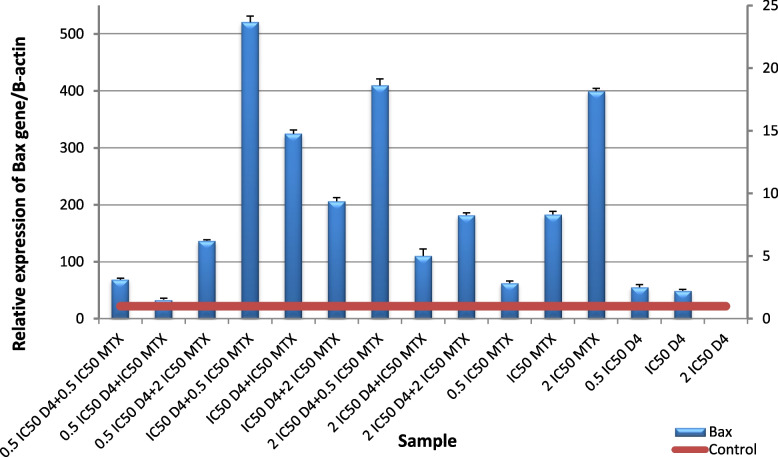
Real-time quantitative PCR analysis of *Bax* expression in untreated HepG2cells, and HepG2 treated by Formula (D4), MTX and their combination ratios

**Fig. 13:**
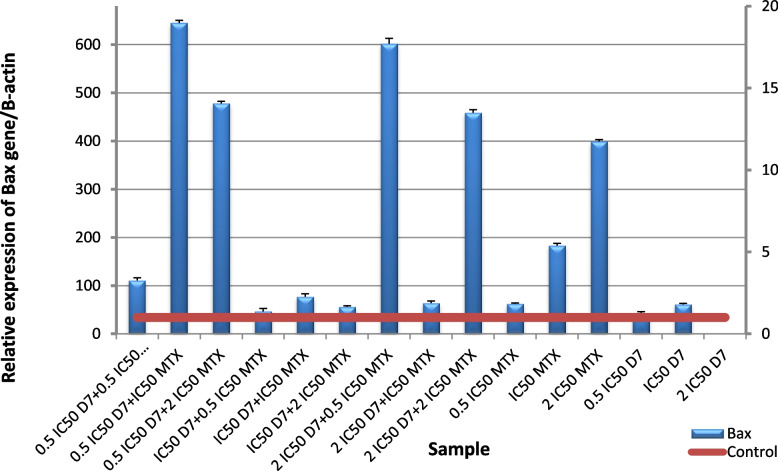
Real-time quantitative PCR analysis of Bax expression in untreated HepG2cells, and HepG2 treated by of Formula (D7), MTX and their combination ratios

**Fig. 14:**
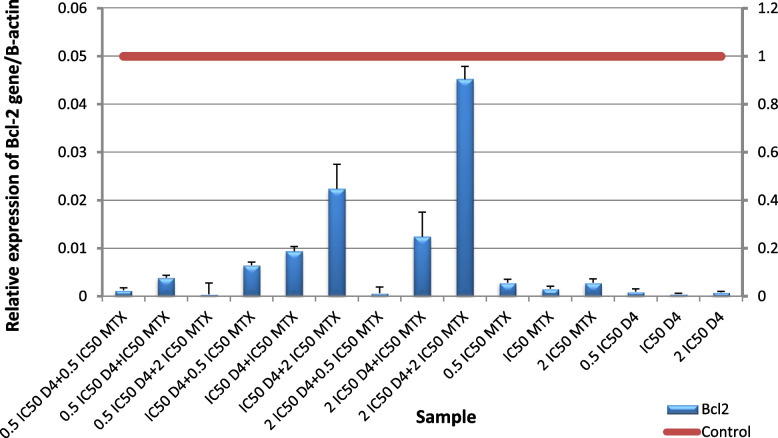
Real-time quantitative PCR analysis of *Bcl-2* expression in untreated HepG2cells, and HepG2 treated by Formula (D4), MTX and their combination ratios

**Fig. 15:**
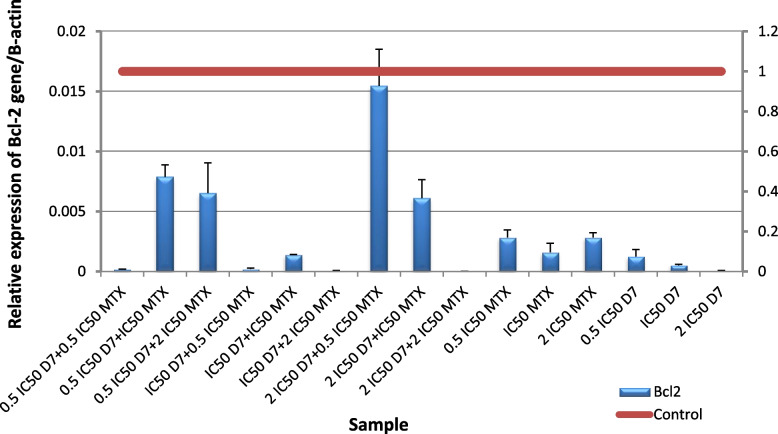
Real-time quantitative PCR analysis of Bcl-2 expression in untreated HepG2cells, and HepG2 treated by of Formula (D7), MTX and their combination ratios

The obtained results revealed a significant (*p* ≤ 0.001) up-regulation of *Bax* gene expression level and downregulation of *Bcl-2* gene expression level in HepG2 cells following administration of (D4), (D7), MTX alone or in combination for 48 h compared to untreated cells. Nano formula D4 has better effect than mixture D7 at all levels proving that nano formulation enhances the effect of the molecules. For *Bax* gene expression level, the highest expression was observed in the cells treated with the ratio (IC50 D4 + 0.5 IC50 MTX), followed by the cells treated with (2 IC50 D4 + 0.5 IC50 MTX) Fig. 12, and for D7 The highest expression was in the cells treated with (IC50 D7 + 0.5 IC50 MTX Fig. 13. For *Bcl-2* gene expression level, the lowest expression was in the cells treated with (0.5 IC50 D4 + 2 IC50 MTX), followed by the cells treated with (2 IC50 D4 + 0.5 IC50 MTX), then (0.5 IC50 D4 + 0.5 IC50 MTX) and finally (2 IC50 D4 + 2 IC50 MTX) Fig. 14. These results were more effective than D7 combination results Fig. 15. From apoptotic gene results we have the best combination ratios for using in further studies (IC50 D4 + 0.5 IC50 MTX).

**Fig. 16:**
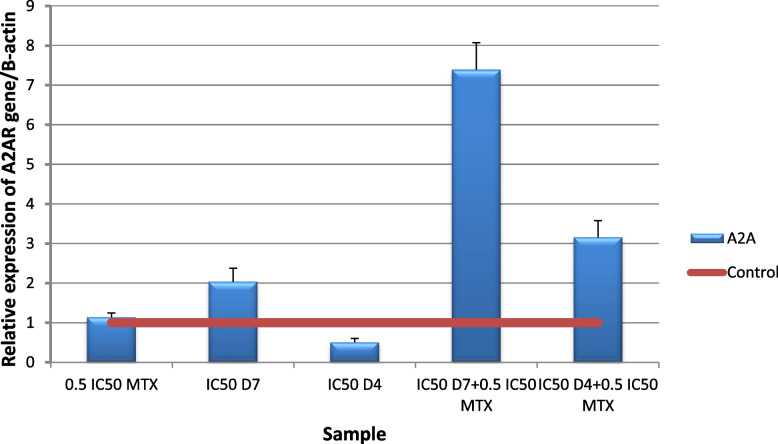
Real-time quantitative PCR analysis of A2AR expression in untreated HepG2cells, and HepG2 treated by of Formula (D4), (D7), MTX and their combination ratios

**Fig. 17:**
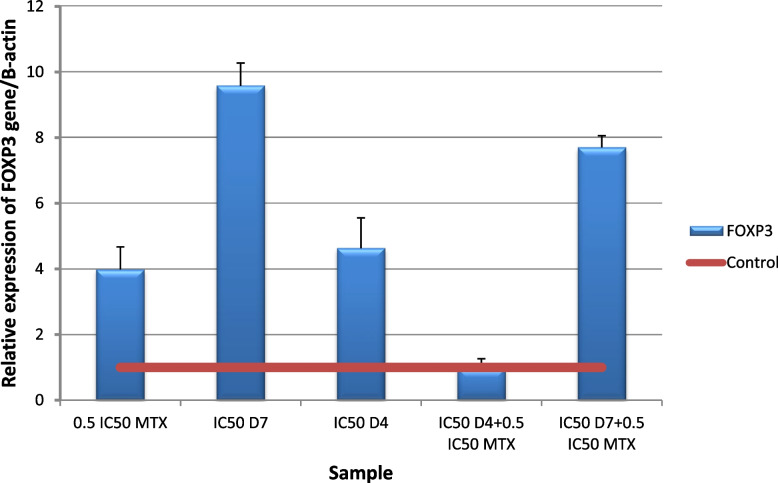
Real-time quantitative PCR analysis of FOXP3 expression in untreated HepG2cells, and HepG2 treated by of Formula (D4), (D7), MTX and their combination ratios

*A2AR gene* expression results revealed a significant (*p* ≤ 0.001) Up regulation in HepG2 cells following administration (IC50D4 + 0.5 IC50MTX) for 48 h compared to untreated cells. The same effect appeared for (IC50D7 + IC50MTX) but nano formula concentration was better. Figures 16 and 17. That is feedback to caffeine present in D4 to block A2A receptor, so its expression has increased as feedback Fig. 16 that agrees with docking results of caffeine. Folic acid had also shown affinity to A2A receptor and may also be a factor to the overexpression of A2A receptor. Novel drugs that block A2AR-adenosinergic effects and/or adenosine generation can increase pathogen destruction, selectively destroy malignant tissues, augment immune clearance of malignant cells and block permissive angiogenesis [14, 116]. Caffeine is a known antagonist of adenosine receptor. Inhibition of these receptors may improve MTX effect [110].

Adenosine binds to A2A receptor, triggering a Gs-protein response and increasing intracellular cAMP. This, in turn, upregulates inhibitory cytokines such as TGF-β and PD-1. The high amounts of TGF-β in the TME promote the survival of cancer cells through the induction of Foxp3 + regulatory T cells and the inhibition of natural killer cell-based anticancer immune responses [17]. *FOXP3 gene* expression level revealed a significant (*p* ≤ 0.001) down regulation in HepG2 cells following administration of (IC50 D4 + 0.5IC50 MTX) for 48 h compared to untreated cells. on the other hand, the ratios revealed a significant (*p* ≤ 0.001) upregulation Fig. 17.

This finding is in agreement with a study by Bao that showed that the levels of Foxp3 were significantly decreased by the adenosine antagonist CSC [117]. Drugs that inhibit the A2AR-mediated adenosine pathway may enhance antitumor immunity by preventing the effects of extracellular adenosine produced from both tissue and Tregs [117].

**Fig. 18:**
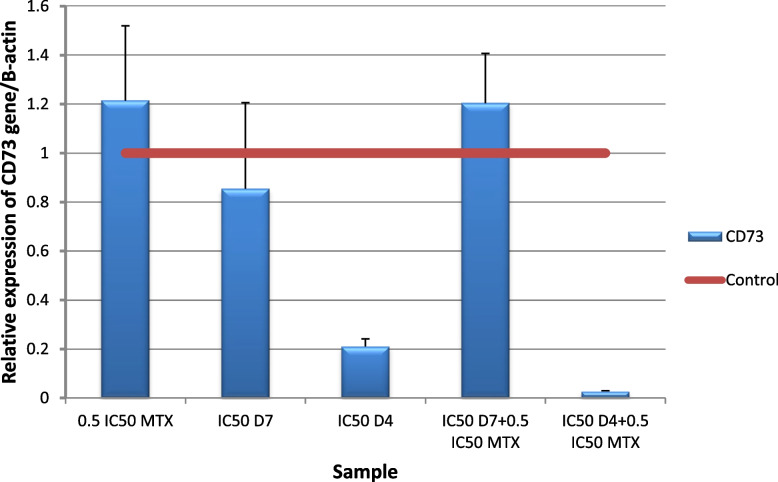
Real-time quantitative PCR analysis of CD73 expression in untreated HepG2cells, and HepG2 treated by of Formula (D4), (D7), MTX and their combination ratios

**Fig. 19:**
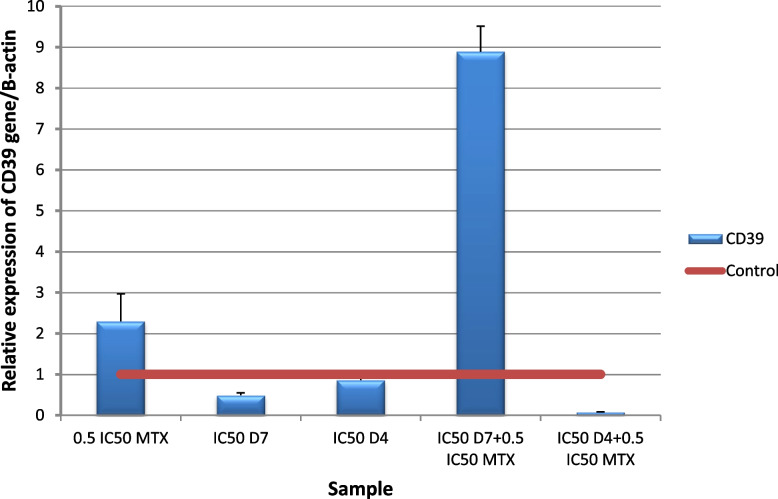
Real-time quantitative PCR analysis of CD39 expression in untreated HepG2cells, and HepG2 treated by of Formula (D4), (D7), MTX and their combination ratios

Our results showed that MTX increased CD73 expression, which is in agreement with Figueiró s study [118]. The results showed a significant (p ≤ 0.001) down regulation of CD39 and CD73 gene expression level in HepG2 cells following the addition of (IC50 D4 + 0.5IC50 MTX) for 48 h compared to untreated cells Figs. 18 and 19. Inhibition of A2aR signaling leads to improved immune activation and anti-tumor activity of MTX in combination.The overexpression of D39 and CD73 in tumors has been linked to a worse prognosis and chemotherapy resistance in patients with various types of cancer [21]. The increased activity of CD39 and CD73 ectoenzymes that produce extracellular adenosine has important pharmacological implications, as most of the immunosuppressive activities can be prevented by extracellular adenosine-degrading, metabolizing or antagonist drugs [119]. Study of Pinna has primarily focused on two aspects of immunosuppressive adenosine: 1) the inhibition of adenosine production in the tumor microenvironment (TME) by targeting CD73 and/or CD39, and 2) the blockade of adenosine signaling by targeting the A2a and A2b receptors. These findings provide a promising direction for future research and development of more effective cancer therapies [120].

Our objective will be to measure anti-inflammatory and pro-inflammatory cytokines, along with related parameters such as regulatory T cells (Tregs) and cyclic adenosine monophosphate (cAMP) [121]. By conducting this investigation, we aim to advance our understanding of immune regulation.

The combination of CAF-FA-CS-NPs (D4) and MTX holds promise for various types of cancers, especially those with overexpression of folate receptors where the targeted drug delivery provided by folic acid (FA) is beneficial. Our study has shown potential in liver and breast cancer cells, demonstrating the effectiveness of this approach. However, to establish its widespread use in cancer treatment and to translate it into the real-world clinical setting, further comprehensive studies and clinical trials are imperative. These future studies will shed light on the safety, efficacy, and optimal application of this combination across different cancer types and stages, paving the way for its integration into routine cancer treatment protocols.

## Conclusions

CAF-FA-CS-NPs were successfully prepared and characterized with good loading capacity for both CAF and FA. The drug release study showed that 50–60% of caffeine and folic acid was released after 3.5 h. The nano formula D4 enhanced the antioxidant and anticancer effect of both caffeine and folic acid. Docking results showed that caffeine and folic acid had the affinity to block the A2A receptor by binding with amino acids in the active site. The nano formula D4 was found to have a better effect than the non-nano mixture D7. Different combination ratios of MTX and D4 were studied to identify the optimal combination for further related genetic studies. The combination of D4 with MTX (IC50D4 + 0.5 IC50MTX) significantly upregulated A2A and downregulated Foxp3, CD73, and CD39 gene expression. This is a novel anticancer formula that enhances the efficacy of methotrexate by reducing its immune suppression effects. Furthermore, its own anticancer effect. This strategy has the potential to improve cancer treatment outcomes and warrants further investigation for use with other chemotherapy agents that have similar limitations. By developing such strategies, we can improve cancer therapies.

## Data Availability

"Data and materials used in this study are available upon request. Please contact the corresponding author for more information."

## Abbreviations

MTX: Methotrexate
CAF: Caffeine
FA: Folic acid
CS: Chitosan
TPP: Sodium tripolyphosphate
A2A: Adenosine 2a Receptor
D4 (CAF-FA-CS-NPs): Caffeine-Folic Acid-Loaded-Chitosan Nanoparticles
D7: Mixture of caffeine and folic acid with the same ratio in D4
